# Effects of microbe-derived antioxidants on growth performance, hepatic oxidative stress, mitochondrial function and cell apoptosis in weaning piglets

**DOI:** 10.1186/s40104-024-01088-3

**Published:** 2024-10-02

**Authors:** Chengbing Yu, Yuxiao Luo, Cheng Shen, Zhen Luo, Hongcai Zhang, Jing Zhang, Weina Xu, Jianxiong Xu

**Affiliations:** Shanghai Key Laboratory of Veterinary Biotechnology, School of Agriculture and Biology, Shanghai Jiao Tong University, Shanghai, 200240 China

**Keywords:** Apoptosis, Microbe-derived antioxidants, Mitochondrial function, Oxidative stress, Weaning piglets

## Abstract

**Background:**

Weaning causes redox dyshomeostasis in piglets, which leads to hepatic oxidative damage. Microbe-derived antioxidants (MA) have great potential for anti-oxidation. This study aimed to investigate changes in hepatic redox system, mitochondrial function and apoptosis after weaning, and effects of MA on growth performance and liver health in weaning piglets.

**Methods:**

This study consisted of 2 experiments. In the both experiments, piglets were weaned at 21 days of age. In Exp. 1, at 21 (W0), 22 (W1), 25 (W4), 28 (W7), and 35 (W14) days of age, 6 piglets were slaughtered at each timepoint. In Exp. 2, piglets were divided into 2 groups: one received MA gavage (MA) and the other received saline gavage (CON). At 25 days of age, 6 piglets from each group were sacrificed.

**Results:**

In Exp. 1, weaning caused growth inhibition and liver developmental retardation from W0 to W4. The mRNA sequencing between W0 and W4 revealed that pathways related to “regulation of apoptotic process” and “reactive oxygen species metabolic process” were enriched. Further study showed that weaning led to higher hepatic content of reactive oxygen species (ROS), H_2_O_2_ and O_2_^−^. Weaning enhanced mitochondrial fission and suppressed their fusion, activated mitophagy, thus triggering cell apoptosis. In Exp. 2, MA improved growth performance of piglets with higher average daily gain (ADG) and average daily feed intake (ADFI). The hepatic ROS, as well as products of oxidative damage malonaldehyde (MDA) and 8-hydroxy-2′-deoxyguanosine (8-OHdG) in the MA group decreased significantly than that of the CON group. The MA elevated mitochondrial membrane potential, increased activity of mitochondrial respiratory chain complexes (MRC) I and IV, enhanced mitochondrial fusion and reduced mitophagy, thus decreasing cell apoptosis.

**Conclusions:**

The present study showed that MA improved the growth performance of weaning piglets and reversed weaning-induced oxidative damage, mitochondrial dysfunction, and apoptosis. Our results suggested that MA had promising prospects for maintaining liver health in weaning piglets and provided a reference for studies of liver diseases in humans.

**Graphical Abstract:**

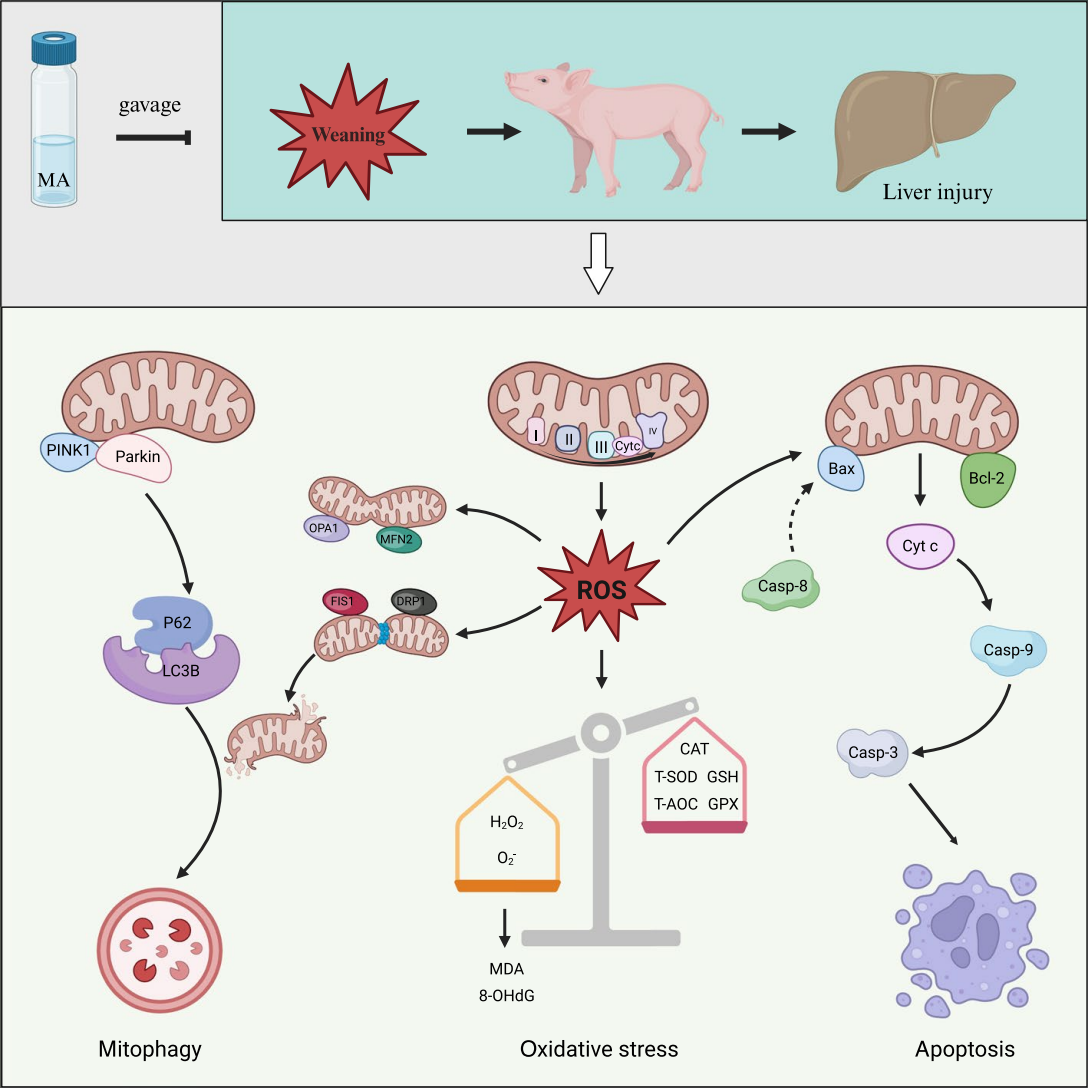

**Supplementary Information:**

The online version contains supplementary material available at 10.1186/s40104-024-01088-3.

## Background

Over the last few decades, based on the progress of nutrition research and the development of intensive farming techniques, piglets have been progressively weaned at 3 to 4 weeks of age [1]. Early weaning improves production efficiency but also leads to poor growth performance, high morbidity and mortality, and finally causes enormous economic losses in the pig industry [2, 3]. It has been proved that early weaning could cause redox dyshomeostasis in piglets [4, 5]. Our previous studies also showed that weaning-induced oxidative stress significantly contributed to intestinal and hepatic damage [6, 7]. Under normal conditions, the redox system is maintained at a sophisticated balance by oxidant and antioxidant responses. Once the balance is broken, reactive oxygen species (ROS), including superoxide anion (O_2_^−^), hydrogen peroxide (H_2_O_2_), and hydroxyl radical (·OH) excessively accumulate. The ROS play an important role in various cellular signaling processes and are toxic to proteins, lipids, and DNA if excessively accumulated in cells [8, 9]. Oxidative stress is considered one of the main factors hindering the health and growth performance of weaning piglets [10].

The liver is an important site for ROS production because of its metabolic and detoxification activities [11]. A shift from redox balance to oxidative stress is considered a pathological mechanism that results in the initiation and progression of various liver diseases, such as viral hepatitis, liver cirrhosis, and hepatocellular carcinoma [11, 12]. Studies have confirmed that weaning slows liver development, impairs liver antioxidant function [13], and induces high ROS levels that trigger apoptosis and autophagy [7]. These studies indicate that weaning is detrimental to liver health and development in piglets. Mitochondria are one of the main sources of endogenous ROS [14]. Mitochondria play pivotal roles in a variety of cellular processes, including ATP generation, programmed cell death, signal transduction, and innate immunity, and their dysfunction has been implicated in various liver diseases [12]. The mitochondrial respiratory chain (MRC) is one of the main providers for mitochondrial ROS, which also makes mitochondria prone to oxidative stress [15, 16]. Compared with suckling piglets, weaning reduces the hepatic activity of MRC complex III (MRC III) and MRC IV [17]. Our previous study also found that weaning can suppress the hepatic content of succinate and ATP [18]. The abnormal metabolism of succinate has been reported to participate in ROS production, inflammatory responses, and metabolic disorders [19–21]. These findings suggest that weaning-induced hepatocyte apoptosis is closely related to redox dyshomeostasis and mitochondrial dysfunction; however, the mechanism remains unknown.

Antioxidants have been widely used to improve the health and growth performance of weaning piglets. The *Rosa roxburghii* and *Hippophae rhamnoides* have high antioxidant activity [22, 23]. Microbe-derived antioxidants (MA) are the products of *Rosa roxburghii* and *Hippophae rhamnoides* fermented by *Bacillus subtilis*, *Lactobacillus*, *Clostridium butyricum* and *Saccharomyces cerevisiae* following extraction, concentration, sterilization, and inactivation processing [24]. The MA is a postbiotic according to the definition proposed by the International Scientific Association of Probiotics and Prebiotics (ISAPP) [25]. In recent years, postbiotics have proven beneficial to human and animal health and have been widely applied in animal production [26, 27]. We used metabolomics to determine the ingredients of MA and found that it mainly contained organic acids and derivatives (15.96%), organic oxygen compounds (10.80%), organoheterocyclic compounds (7.04%), lipids and lipid-like molecules (6.10%), and others [24]. Our previous studies found that MA exhibits great potential for anti-oxidation and anti-inflammation in vitro [24, 28]. In mice, MA mitigates hepatic oxidative stress caused by a high-fat diet [29, 30]. In piglets, MA protects the intestinal barrier function from weaning stress [6]. However, the effects of MA on the liver of weaning piglets remain unknown. Thus, the present study aimed to investigate the changes of the hepatic redox system, mitochondrial function, and apoptosis after weaning, as well as to evaluate the potential protective effects of MA on weaning piglets’ liver.

## Methods

### Animal management and experiment design

This study was conducted under the guidance of Animal Care and Use Committee of Shanghai Jiao Tong University (approval No. 202201188).

In Exp. 1, 60 crossbred piglets (Duroc × Landrace × Yorkshire) were randomly selected (6 litters, 10 per litter). Each litter represented a replicate and was housed in a separate pen with 10 piglets. All piglets were weaned at 21 days of age and were fed the same experimental diet. At 21 (W0), 22 (W1), 25 (W4), 28 (W7), and 35 (W14) days of age, 6 piglets were slaughtered at each timepoint. The 6 piglets consisted of one piglet from each litter, 3 males and 3 females. This experiment was conducted at a specialized pig farm. During the experiment, all piglets were housed in plastic-floored pens, where the temperature and humidity were maintained at 25 °C and 60%, respectively. Each pen was equipped with a feeder and a nipple drinker. The experimental diets were formulated according to swine nutrition requirements recommended by the National Research Council [31]. The dietary composition and nutrient levels were shown in Table 1 [18].

**Table 1:** Dietary composition and nutrient level (%, as-fed basis)

Item	Content
Ingredients	
Corn	33.00
Extruded corn	22.00
Fermented soybean meal	11.20
Extruded soybean	8.00
Soybean meal	9.40
Fish meal	4.00
Whey powder	6.00
Soybean oil	1.40
White granulated sugar	1.35
Choline chloride	0.20
CaHPO_4_	1.20
Limestone	0.40
NaCl	0.30
L-Lys	0.33
DL-Met	0.12
L-Thr	0.10
Premix^a^	1.00
Total	100.00
Calculated nutrient level	
Metabolizable energy, MJ/kg	14.62
Crude protein	20.50
Crude fat	4.37
Crude fiber	2.34
Crude ash	5.80
Lys	1.17
Met + Cys	0.69
Thr	0.73
Try	0.19
Calcium	0.60
Total phosphorus	0.60

^a^Premix provided the following per kilogram of diet: vitamin A, 3,500 IU; vitamin C, 100 mg; vitamin D_3_, 350 IU; vitamin E, 30 IU; vitamin K, 0.8 mg; vitamin B_1_, 2.0 mg; vitamin B_2_, 6.0 mg; vitamin B_3_, 12.5 mg; vitamin B_6_, 3.0 mg; vitamin B_12_, 0.03 mg; choline chloride, 800 mg; D-pantothenic acid, 15.0 mg; folic acid, 0.45 mg; nicotinic acid, 22.5 mg; biotin, 0.08 mg; Cu (CuSO_4_·5H_2_O), 9.0 mg; Fe (FeSO_4_·H_2_O), 150 mg; Zn (ZnSO_4_·H_2_O), 150 mg; Mn (MnSO_4_·5H_2_O), 50 mg; I (KI) 0.25 mg; Se (Na_2_SeO_3_·H_2_O), 0.45 mg

In Exp. 2, another new 60 crossbred piglets (Duroc × Landrace × Yorkshire) were randomly selected (6 litters, 10 per litter). Piglets were divided into CON and MA groups, each group had 30 piglets (6 litters, 5 per litter). Each litter represented a replicate and was housed in a separate pen with 5 piglets. The CON group received saline gavage and the MA group received MA gavage. For oral gavage, a V-trough was used to restrain piglets. The mouth was checked for the presence of any foreign matter and the head was tilted up. A mouth gag was inserted into the mouth and a catheter was then put into the mouth. The MA and saline were respectively injected using syringes through the catheter into the mouth [32]. The gavage was carried out at 08:00 and the dosage was 0.4 mL/kg body weight, according to our previous study [30]. The MA (trade name: KB-120, liquid) was provided by Shanghai Jiang Han Biotechnology. The gavage experiment was carried out every day from 19 to 41 days of age. All piglets were weaned at 21 days of age. At 25 days of age, 6 piglets from each group (12 piglets in total) were randomly selected and slaughtered. In each group, the 6 piglets consisted of one piglet from each litter, 3 males and 3 females. The remaining piglets were fed until 41 days of age, on which day their body weight and feed intake were recorded. Experiments 1 and 2 were conducted in the same pig farm. The management and experimental diets in Exp. 2 were the same as those in Exp. 1.

### Sample collection

In Exp. 1, blood and liver samples were collected at each timepoint at 21, 22, 25, 28, and 35 days of age. In Exp. 2, blood and liver samples of the CON and MA groups were collected at 25 days of age. Briefly, fasting blood samples were collected from the precaval vein of piglets after anesthetization with sodium pentobarbital (40 mg/kg body weight). When left at room temperature for 2 h, the blood samples were centrifuged (3,000 × *g*, 10 min, 4 °C) to obtain serum. The piglets were then slaughtered by jugular bloodletting. The complete liver was isolated and weighed. Afterwards, the fresh liver tissue was cut into small pieces to prepare pathological sections. The serum and remaining liver tissue were immediately stored at –80 °C for further analysis.

### Growth performance

In Exp. 2, at 21, 25, and 41 days of age, the body weight and feed intake of piglets in each pen were recorded. The hepatosomatic index (HSI), average daily gain (ADG) and average daily feed intake (ADFI) were calculated as follows:

$$\mathrm{HSI}\;\left(\%\right)=\mathrm{(liver}\;\mathrm{weight}\;\div\mathrm{body}\;\mathrm{weight)}\times100\%$$

$$\mathrm{ADG}\;\left(\mathrm g/\mathrm d\right)=\mathrm{(total}\;\mathrm{weight}\;\mathrm{gain }\;\div\mathrm{number}\;\mathrm{of}\;\mathrm{piglets)}\div\mathrm{test}\;\mathrm{days}$$

$$\mathrm{ADFI}\;\left(\mathrm g/\mathrm d\right)=\mathrm{(total}\;\mathrm{feed}\;\mathrm{intake}\div\mathrm{number}\;\mathrm{of}\;\mathrm{piglets)}\div\mathrm{test}\;\mathrm{days}$$

### H&E staining

Fresh liver tissues were fixed in 4% paraformaldehyde for 48 h, embedded in paraffin, and cut into 4 μm sections. The sections were then stained with hematoxylin–eosin (H&E). All staining procedures were performed by Runnerbio Technology Co., Ltd. (Shanghai, China). Briefly, the sections were deparaffinized, rehydrated to distilled water, stained with hematoxylin for 10 min, and washed with tap water. Cleared sections were stained with alcohol-eosin for 30 s and then dehydrated in ascending alcohol solutions (2 changes of 95%, 2 changes of 100%, 30 s for each change). Subsequently, the sections were cleared in xylene carbonate for 30 s and 2 changes of xylene for 30 s. Finally, the sections were mounted with neutral resin and coverslips for microscopic observation.

### TUNEL assay

Hepatic apoptosis was determined with One-step Terminal Deoxynucleotidyl Transferase-mediated dUTP Nick-end Labeling (TUNEL) assay. Firstly, the paraffin sections of liver tissues were deparaffinized and rehydrated. Then the sections were incubated with 20 µg/mL proteinase K for 15 min and treated with 3% H_2_O_2_ for 5 min at room temperature. Next, the sections were incubated with the TUNEL reagents in the dark at 37 °C for 1 h followed by staining with 4′-6-diamidino-2-phenylindole (DAPI). Finally, the sections mounted with coverslips were observed (Nikon Eclipse 50i) and pictured (Nikon DS-Fil, Tokyo, Japan).

### Transmission electron microscope

The ultrastructure of liver tissue was observed using transmission electron microscopy (TEM). The main operational steps were as follows: (1) Pre-fixation: fresh tissues in 2.5% glutaraldehyde at 4 °C overnight. (2) Rinse: ice-cold PBS for 15 min, three times. (3) Post-fixation: 1% osmium tetroxide at 4 °C for 2 h. (4) Dehydration: gradient ethanol solutions, 50%, 70%, 80%, and 90% at 4 °C for 15 min (70% for overnight); 100% ethanol solutions, 2 times for 15 min each at room temperature. (5) Substitution: acetone for 15 min, twice at room temperature; 2:1 acetone and epoxy resin for 1 h at room temperature; 1:2 acetone and epoxy resin for 4 h at room temperature; epoxy resin 2 times for 12 h each. (6) Embedding: epoxy resin for 48 h at 65 °C. (7) Sectioning: cutting into 70 nm sections with ultramicrotome (Leica EM UC7, Wetzlar, Germany). (8) Staining: uranyl acetate for 10 min, followed by lead acetate for 10 min at room temperature. (9) Observation: observation and recording using a TEM (JEM-1230, Joel, Japan).

### Dihydroethidium staining

The dye dihydroethidium (DHE, Yeasen, China) was used to assess ROS levels in liver tissue. Fresh liver tissues were fixed in 4% paraformaldehyde for 24 h and dehydrated in 20% and 30% sucrose solutions. The liver tissues were immersed in optimal cutting temperature compound (OCT) for 2 h, 4 h, and 6 h at room temperature. The OCT was replaced with fresh one each time. The processed samples were frozen in a cryostat (–20 °C) and then cut into 10 µm sections using a microtome. The sections were placed on slides, circled with a hydrophobic barrier pen, and incubated with an autofluorescence quencher. The DHE and DAPI were used for staining. Subsequently, the slides were incubated with antifade mounting medium and mounted with coverslips. Finally, the slides were observed (Nikon Eclipse 50i) and photographed (Nikon DS-Fil, Tokyo, Japan).

### Detection of biochemical parameters

To evaluate the hepatic redox state of weaning piglets, we detected hepatic ROS, including superoxide anion (O_2_^−^) and hydrogen peroxide (H_2_O_2_); antioxidant parameters, including total superoxide dismutase (T-SOD), catalase (CAT), glutathione peroxidase (GPX), glutathione (GSH), and total antioxidant capacity (T-AOC); and oxidative damage product, including malondialdehyde (MDA), protein carbonyl (PC), and 8-hydroxy-2′-deoxyguanosine (8-OHdG). Briefly, the liver tissues were homogenized and the supernatant was obtained after centrifugation (3,000 × *g*, 10 min, 4 °C) for further analysis. All detections were conducted according to the manufacturer’ instructions (Nanjing Jiancheng Bioengineering Institute, Nanjing, China) as described previously [7]. Particularly, the homogenate was centrifuged at 600 × *g* (10 min, 4 °C), and the supernatant was centrifuged at 11,000 × *g* (10 min, 4 °C) to obtain mitochondria. The mitochondrial membrane potential (MMP) was measured with 5,5′,6,6′-tetrachloro-1,1′,3,3′-tetraethylbenzimi-dazoylcarbocyanine iodide (JC-1) dye. The obtained mitochondria were disrupted by ultrasonication (power 200 W, ultrasonication 5 s, interval 15 s, 15 cycles), and the activity of mitochondrial respiratory chain complexes was determined as previously described [18]. Optical density was measured with a microplate reader (SYNERGY 2, BioTek, Winooski, VT, USA) and a spectrophotometer (L6 Split Beam, INESA, Shanghai, China).

### Western blot

The expression of target proteins was detected by Western blot as previously described [33]. Briefly, the liver samples were homogenized in radio-immunoprecipitation assay (RIPA) lysis buffer (Beyotime, Shanghai, China) and centrifuged (12,000 × *g*, 20 min, 4 °C) to collect the supernatant. The protein concentration was determined and mixed with loading buffer before thermal denaturation. Samples were electrophoresed on SDS-PAGE gels and transferred to polyvinylidene difluoride (PVDF, Millipore, Billerica, MA, USA) membranes. The membranes were incubated with blocking buffer for 2 h at room temperature and then incubated with primary antibodies overnight at 4 °C. The next day, the membranes were incubated with anti-rabbit or anti-mouse IgG, HRP-linked antibodies for 2 h. Images were acquired using an enhanced chemiluminescence detection system (Tanon, Shanghai, China). ImageJ software [34] was used to quantify the density of the specific protein bands. The antibodies used in this study were listed in Table S1 (Additional file 1).

### mRNA sequencing

Total RNA was extracted from liver tissues with Total RNA Kit (Omega Bio-Tek, Norcross, GA, USA). The RNA concentration was determined with NanoDrop Lite Spectrophotometer (Thermo Fisher Scientific, Waltham, MA, USA). The purity and integrity were evaluated by agarose gel electrophoresis and capillary electrophoresis using an Agilent 2100 Bioanalyzer (Agilent, Palo Alto, CA, USA). The mRNA was purified from the total RNA with oligo dT magnetic beads and fragmented into 200–300 bp. The mRNA fragments were reverse-transcribed to the first strand of cDNA in the presence of random hexamer primers and reverse transcriptase, and then double-stranded cDNA (ds cDNA) was synthesized based on the first strand of cDNA. The base T was replaced by base U during the second strand of cDNA synthesis. After 5′ end repair and 3′ add A tail, the purified ds cDNA was added with adapters. Library fragments were amplified by PCR and fragments of approximately 450 bp were selected. Paired-end sequencing was performed using Next-Generation Sequencing based on the Illumina HiSeq sequencing platform.

The raw data obtained by sequencing were further filtered to remove adaptors and low-quality reads. Subsequently, the reads were mapped to the reference genome using online software HISAT2 (http://ccb.jhu.edu/software/hisat2/index.shtml). HTSeq was used to compare the read count value of each gene as the original expression of the gene, and the number of fragments per thousand bases from a gene per million fragments (FPKM) mapped values were used to standardize the quantification of the genes. The DESeq software was used to analyze the differentially expressed genes (DEGs). Two criteria |log_2_foldchange| > 1 and *P*-value < 0.05, were adopted to screen for DEGs between the control and the sample groups. The Gene Ontology (GO) and Kyoto Encyclopedia of Genes and Genomes (KEGG) pathway enrichment of DEGs were analyzed by Blast2go and KAAS software, respectively. The main biological functions of the DEGs were determined by enrichment analysis of metabolic pathways. Transcriptomics was performed by Shanghai Personalbio Technology Co., Ltd. (Shanghai, China).

### Statistical analysis

In Exp. 1, for comparison between the 5 groups, one-way ANOVA and Tukey’s HSD post-hoc test or Kruskal–Wallis test and Dunn’s post-hoc test were employed. In Exp. 2, for comparison between the 2 groups, t-test or Mann–Whitney U test were employed. All data were presented as mean ± SEM, and difference was considered statistically significant when *P* < 0.05 (^*^P < 0.05, ^**^P < 0.01). Analyses were performed using SPSS 20.0 (IBM Corporation, Armonk, NY, USA). All figures were graphed by GraphPad Prism 9.0 (GraphPad Software, La Jolla, CA, USA).

## Results

### Weaning induced developmental retardation of piglet liver

**Fig. 1:**
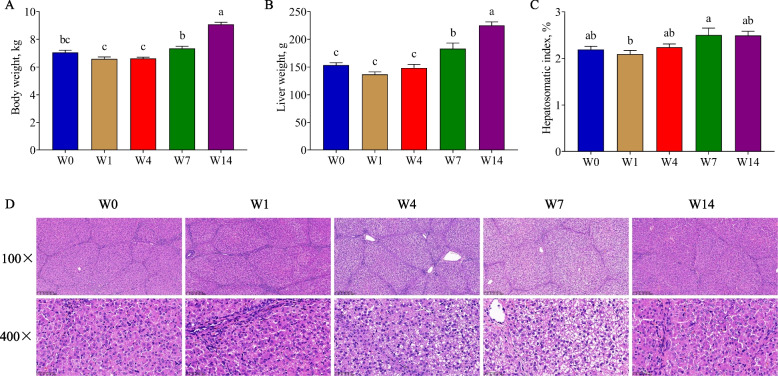
The body and liver weight, hepatosomatic index and H&E staining. **A**–**C** Body weight, liver weight and hepatosomatic index, respectively; **D** H&E staining of liver tissues. W0, W1, W4, W7, and W14 respectively represented 21, 22, 25, 28, and 35 days of age. Data were presented as mean ± SEM (*n* = 6). Values with different letters differ significantly (*P* < 0.05)

In Exp. 1, the body and liver weight of piglets showed growth inhibition from W0 to W4 and increased from W4 to W14 (*P* < 0.05, Fig. 1A and B). The hepatosomatic index on W7 was significantly higher than that on W1 (*P* < 0.05, Fig. 1C). The H&E staining (Fig. 1D) showed that the hepatic lobules had a complete structure, clear boundaries, and normal cell nucleus morphology on W0. On W4 and W7, the boundaries of the hepatic lobules were indistinct, and the hepatocytes presented obvious cytoplasmic vacuolation and nuclear shrinkage, indicating that apoptosis might occur. On W14, the cell morphology gradually became normal.

### Analysis of mRNA sequencing

**Fig. 2:**
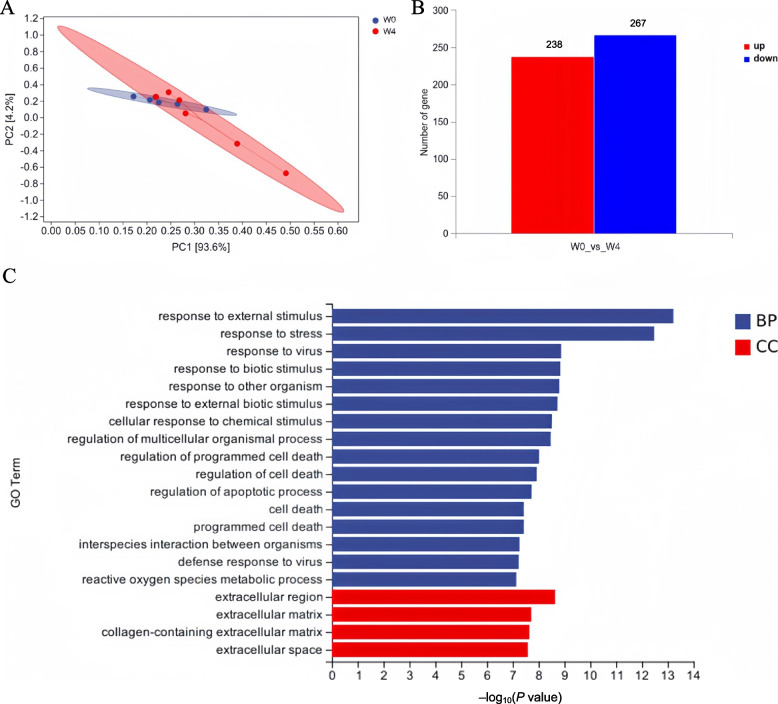
The results of mRNA sequencing. **A** The PCA analysis, different colors represented different groups and one circle represents one sample; **B** Differential expression analysis, the numbers meant the up-regulated and down-regulated genes, respectively; **C** Enrichment analysis, the *Y*-axis represented the GO terms and the *X*-axis represented –log_10_(*P* value). BP: biological process; CC: cellular component. The W0 represented the weaning day and W4 represented 4 days after weaning (*n* = 6)

When observing the growth inhibition from W0 to W4, the mRNA sequencing was employed to analyze hepatic differential genes between W0 and W4. The results showed that the samples of W0 and W4 were clearly separated in PCA analysis (Fig. 2A). Compared with W0, 238 genes were up-regulated and 267 genes were down-regulated on W4 (Fig. 2B). The enriched GO terms were mostly related to “response to stimulus” and “regulation of cell death”. Notably, the “reactive oxygen species metabolic process” was significantly enriched (Fig. 2C).

### Weaning resulted in oxidative damage

**Fig. 3:**
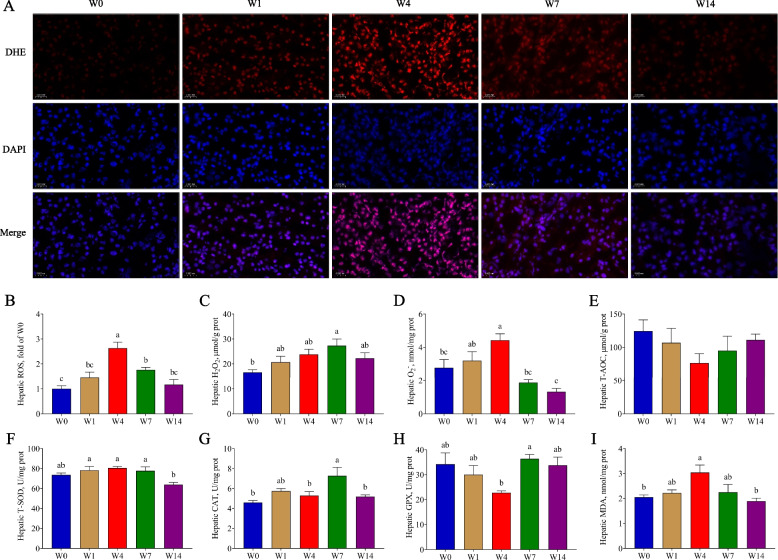
The hepatic redox parameters. **A** and **B** DHE Staining with frozen liver sections and fluorescence intensity of ROS; **C**–**E** Hepatic H_2_O_2_, O_2_^−^ and T-AOC content; **F**–**H** Hepatic T-SOD, CAT and GPX activity; **I** Hepatic MDA content. W0, W1, W4, W7, and W14 respectively represented 21, 22, 25, 28, and 35 days of age. Data were presented as mean ± SEM (ROS, *n* = 3; others, *n* = 6). Values with different letters differ significantly (*P* < 0.05). ROS: Reactive oxygen species; T-AOC: Total antioxidant capacity; T-SOD: Total superoxide dismutase; CAT: Catalase; GPX: Glutathione peroxidase; MDA: Malonaldehyde

The mRNA sequencing revealed that the “reactive oxygen species metabolic process” was enriched between W0 and W4. Therefore, we further investigated hepatic redox parameters. The hepatic ROS content increased from W0 to W4 and then gradually decreased from W4 to W14 (*P* < 0.05, Fig. 3A and B). The hepatic H_2_O_2_ and O_2_^−^ content on W7 and W4 were significantly higher than those on W0 (*P* < 0.05, Fig. 3C and D). No significant difference in T-AOC content was observed after weaning (*P* > 0.05, Fig. 3E). The T-SOD activity on W14 was significantly lower than that on W1, W4, and W7 (*P* < 0.05, Fig. 3F). The CAT activity on W7 was significantly higher than that on W0 (*P* < 0.05, Fig. 3G). The GPX activity gradually decreased from W0 to W4 and increased thereafter (*P* < 0.05, Fig. 3H). The MDA showed an upward trend from W0 to W4 and then declined from W4 to W14 (*P* < 0.05, Fig. 3I).

### Weaning caused mitochondrial dysfunction

**Fig. 4:**
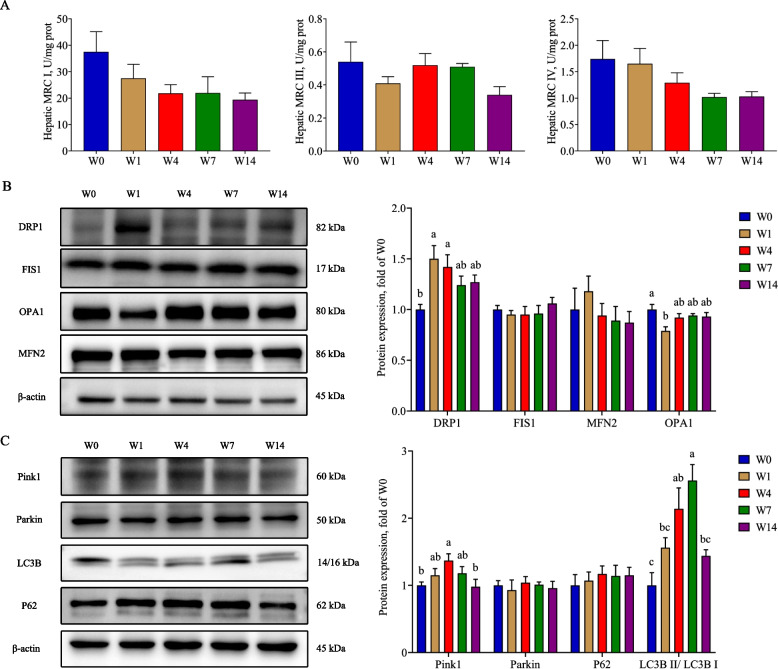
The effects of weaning on mitochondrial function. **A** Activity of mitochondrial respiratory chain complexes; **B** The expression of proteins related to mitochondrial fusion and fission; **C** The expression of proteins related to mitophagy. W0, W1, W4, W7, and W14 respectively represented 21, 22, 25, 28, and 35 days of age. Data were presented as mean ± SEM (*n* = 6). Values with different letters differ significantly (*P* < 0.05). MRC I: Mitochondrial respiratory chain complex I; MRC III: Mitochondrial respiratory chain complex III; MRC IV: Mitochondrial respiratory chain complex IV; DRP1: Dynamin-related protein 1; MFN2: Mitofusin 2; FIS1: Fission protein 1; OPA1: Optic atrophy protein 1; Pink1: PTEN induced putative kinase 1; Parkin: E3 ubiquitin ligase; P62: Sequestosome 1; LC3B: Microtubule associated protein 1 light chain 3 beta

The mitochondria are one of the main sources of endogenous ROS. We further investigated the activity of MRC, mitochondrial fusion and fission, and mitophagy to evaluate mitochondrial function. There was no statistical difference in activity of MRC I, III, and IV during the experiment (*P* > 0.05, Fig. 4A). We further investigated the expression of proteins related to mitochondrial function with Western blot assay. The DRP1 expression on W1 and W4 were significantly higher than that on W0 (*P* < 0.05), and OPA1 expression was significantly lower on W1 than that on W0 (*P* < 0.05, Fig. 4B). The expression of Pink1 elevated significantly from W0 to W4 and declined from W4 to W14 (*P* < 0.05, Fig. 4C). The ratio of LC3B II to LC3B I increased gradually from W0 to W7 and decreased since then (*P* < 0.05). There were no obvious differences in FIS1, MFN2, Parkin, and P62 during the experiment (*P* > 0.05).

### Weaning triggered apoptosis

**Fig. 5:**
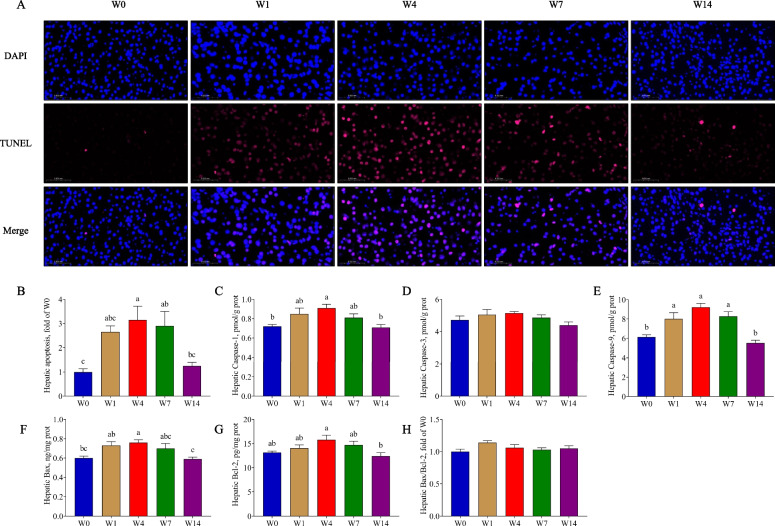
The effects of weaning on cell apoptosis. **A** and **B** TUNEL staining and apoptotic ratio; **B**–**G** Hepatic content of Caspase-1, Caspase-3, Caspase-9, Bax and Bcl-2; **H** Ratio of Bax to Bcl-2. W0, W1, W4, W7, and W14 respectively represented 21, 22, 25, 28, and 35 days of age. Data were presented as mean ± SEM (apoptotic ratio, *n* = 3; others, *n* = 6). Values with different letters differ significantly (*P* < 0.05). Caspase-1: Cysteine aspartate-specific protease 1; Caspase-3: Cysteine aspartate-specific protease 3; Caspase-9: Cysteine aspartate-specific protease 9; Bcl-2: B-cell lymphoma 2; Bax: Bcl-2 associated X protein

The mitochondrial damage is closely associated with apoptosis. The TUNEL assay showed that cell apoptosis increased from W0 to W4 and then gradually decreased from W4 to W14 (*P* < 0.05, Fig. 5A and B). The hepatic content of Caspase-1, Caspase-9, Bcl-2, and Bax showed a similar tendency, which increased from W0 to W4 and then decreased from W4 to W14 (*P* < 0.05, Fig. 5C and E–G). The Caspase-3 content and ratio of Bax to Bcl-2 showed no difference in statistics after weaning (*P* > 0.05, Fig. 5D and H).

### MA improved growth performance of piglets

**Fig. 6:**
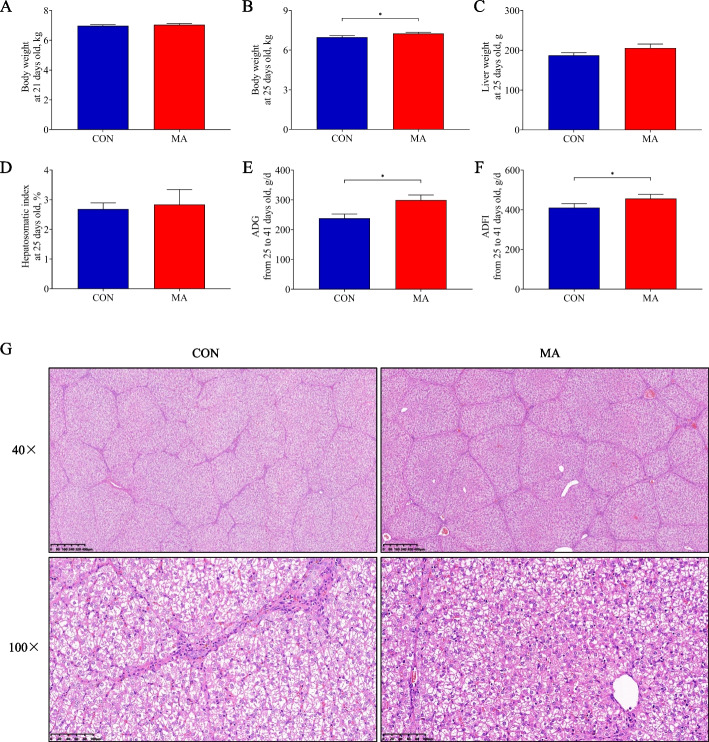
The effects of MA on piglet growth performance. **A** and **B** Body weight of piglets at 21 and 25 d old, respectively; **C** Liver weight of piglets at 25 d old; **D** Hepatosomatic index of piglets at 25 d old; **E** and **F** ADFI and ADG of piglets from 25 to 41 d old; **G** H&E staining of piglet liver. CON: piglets gavaged saline; MA: piglets gavaged MA. Data were presented as mean ± SEM (*n* = 6). *: *P* < 0.05; **: *P* < 0.01. ADG: Average daily gain; ADFI: Average daily feed intake

In Exp. 2, the body weight of the MA group showed no statistical difference at 21 days of age (*P* > 0.05, Fig. 6A) but significantly increased at 25 days of age compared to the CON group (*P* < 0.05, Fig. 6B). There was no statistical difference in liver weight and hepatosomatic index at 25 days of age between the CON and MA groups (*P* > 0.05, Fig. 6C and D). The ADG and ADFI from 25 to 41 days of age in the MA group were significantly higher than those in the CON group (*P* < 0.05, Fig. 6E and F). The H&E staining showed that the hepatic lobules of the MA group had a complete structure, clear boundaries, and normal cell nucleus morphology compared to those of the CON group. The hepatocytes of the CON group presented obvious cytoplasmic vacuolation compared to those of the MA group (Fig. 6G).

### MA alleviated the oxidative damage

**Fig. 7:**
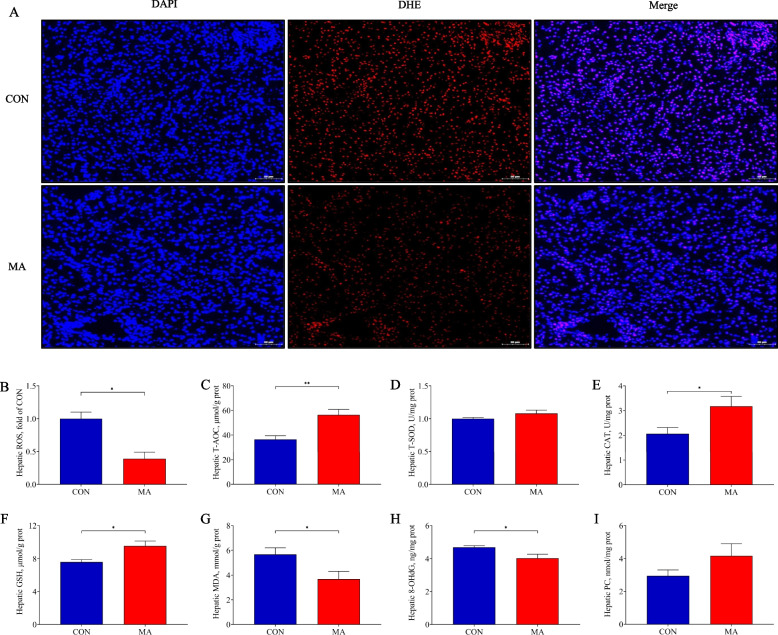
The effects of MA on parameters of redox system in liver tissue. **A** and **B** DHE staining with frozen liver sections and fluorescence intensity of ROS; **C** Content of T-AOC; **D** and **E** Activity of T-SOD and CAT; **F** to **I** Content of GSH, MDA, 8-OHdG, PC. CON: piglets gavaged saline; MA: piglets gavaged MA. Data were presented as mean ± SEM (ROS, *n* = 3; others, *n* = 6). *: *P* < 0.05; **: *P* < 0.01. ROS: Reactive oxygen species; T-AOC: Total antioxidant capacity; T-SOD: Total superoxide dismutase; CAT: Catalase; GSH: Glutathione; MDA: Malonaldehyde; 8-OHdG: 8-hydroxy-2’-deoxyguanosine; PC: Protein carbonyl

The hepatic ROS content was significantly lower in the MA group than that in the CON group (*P* < 0.05, Fig. 7A and B). Compared to the CON group, the T-AOC content increased significantly in the MA group (*P* < 0.05, Fig. 7C). The antioxidant enzyme T-SOD activity showed no statistical difference between the 2 groups (*P* > 0.05, Fig. 7D). The CAT activity and GSH content were significantly higher in the MA group than that in the CON group (*P* < 0.05, Fig. 7E and F). As products of oxidative damage, the MDA and 8-OHdG content significantly decreased in the MA group compared with the CON group (*P* < 0.05, Fig. 7G and H). There was no obvious difference in the PC content between the 2 groups (*P* > 0.05, Fig. 7I).

### MA ameliorated mitochondrial dysfunction

**Fig. 8:**
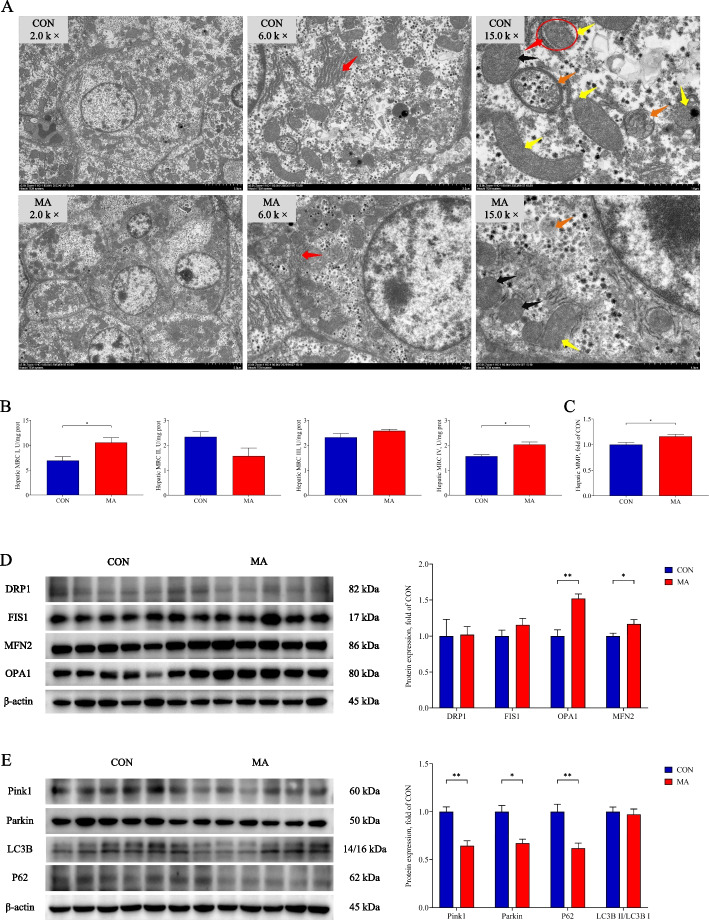
The effects of MA on mitochondrial function. **A** The represented images of mitochondrial ultrastructure with TEM. Endoplasmic reticulum (red arrow); Autolysosome (orange arrow); Damaged mitochondria (yellow arrow); Normal mitochondria (black arrow). **B** Activity of mitochondrial respiratory chain complexes; **C** Hepatic MMP; **D** The expression of proteins related to mitochondrial fusion and fission; **E** The expression of proteins related to mitophagy. CON: piglets gavaged saline; MA: piglets gavaged MA. Data were presented as mean ± SEM (*n* = 6). *: *P* < 0.05; **: *P* < 0.01. MRC I: Mitochondrial respiratory chain complex I; MRC II: Mitochondrial respiratory chain complex II; MRC III: Mitochondrial respiratory chain complex III; MRC IV: Mitochondrial respiratory chain complex IV; MMP: mitochondrial membrane potential; DRP1: Dynamin-related protein 1; MFN2: Mitofusin 2; FIS1: Fission protein 1; OPA1: Optic atrophy protein 1; Pink1: PTEN induced putative kinase 1; Parkin: E3 ubiquitin ligase; P62: Sequestosome 1; LC3B: Microtubule associated protein 1 light chain 3 beta

The ultrastructure of hepatocytes was observed with transmission electron microscope. The results showed that the endoplasmic reticulum (ER, red arrow) of the MA group presented a more regular arrangement than that of the CON group. The damaged mitochondria showed rupture of mitochondrial membrane, as well as loss of cristae and matrix. Compared with the MA group, the CON group had more damaged mitochondria (yellow arrow), which were clearly observed encased in detached ER (red circle) at high magnification (Fig. 8A). The MA significantly increased the activity of MRC I and MRC IV, as well as MMP compared with the CON group (*P* < 0.05, Fig. 8B and C). In Western blot analysis, the expression of MFN2 and OPA1 was higher in the MA group than that in the CON group (*P* < 0.05, Fig. 8D). The expression of Pink1, Parkin, and P62 was significantly lower in the MA group than that in the CON group (*P* < 0.05, Fig. 8E).

### MA reduced cell apoptosis

**Fig. 9:**
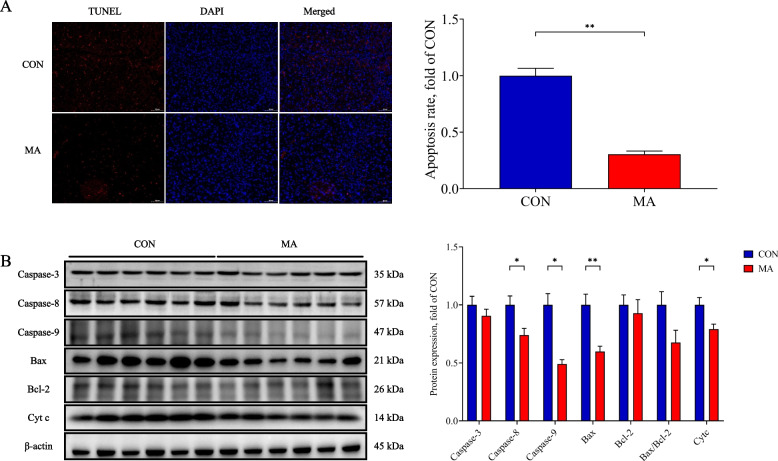
The effects of MA on cell apoptosis. **A** TUNEL staining and apoptotic ratio; **B** Expression of proteins related to apoptosis. CON: piglets gavaged saline; MA: piglets gavaged MA. Data were presented as mean ± SEM (apoptotic ratio, *n* = 3; others, *n* = 6). *: *P* < 0.05; **: *P* < 0.01. Caspase-3: Cysteine aspartate-specific protease 3; Caspase-8: Cysteine aspartate-specific protease 8; Caspase-9: Cysteine aspartate-specific protease 9; Bcl-2: B-cell lymphoma 2; Bax: Bcl-2 associated X protein; Cyt c: Cytochrome c

We further studied effects of MA on cell apoptosis with Western blot assay. The results showed that MA significantly decreased hepatocyte apoptosis compared to that in weaning piglets (*P* < 0.05, Fig. 9A). The expression of Caspase-8, Caspase-9, Bax, and Cyt c decreased significantly in the MA group compared with that in the CON group (*P* < 0.05, Fig. 9B). There was no statistical difference in Caspase-3, Bcl-2 and Bax/Bcl-2 between the 2 groups (*P* > 0.05, Fig. 9B).

## Discussion

Oxidative stress is one of the main factors that hinder the health and growth performance of weaning piglets. Antioxidant additives have been widely used in the pig industry as a promising strategy. Our previous studies found that MA exhibits great potential for anti-oxidation and anti-inflammation [24, 28]. In weaning piglets, MA alleviates oxidative stress and improves intestinal barrier function [6]. The present study showed that weaning caused hepatic oxidative damage, mitochondrial dysfunction, and cell apoptosis. The MA improved the growth performance of piglets and mitigated the weaning-induced oxidative stress and mitochondrial dysfunction, thus reducing cell apoptosis and enhancing liver health of weaning piglets.

Under physiological conditions, the redox system is maintained at a sophisticated balance through pro-oxidant and anti-oxidant responses. In the case of oxidative stress, excessive accumulation of ROS triggers an immune response, metabolic disorders, and cell death, thereby causing or aggravating diseases [35, 36]. Numerous studies have reported that weaning causes redox dyshomeostasis in mammals, resulting in oxidative damage to organs and tissues [37–39]. As a primary organ, the liver plays a pivotal anti-stress role and is highly susceptible to external stimuli, which can readily result in structural and functional damage [11, 12]. The mRNA sequencing revealed that the “reactive oxygen species metabolic process” and “regulation of apoptotic process” were significantly enriched on W4 compared to W0, which was similar to the research on small intestine in weaning piglets [40]. Further research found that weaning increased hepatic ROS level and MDA content, which was in accordance with our previous study [7]. Mitochondria are a major source of endogenous ROS, and their dysfunction contributes significantly to apoptosis. In mitochondrial apoptosis, the caspase activation is closely linked to mitochondrial outer membrane permeabilization (MOMP) [41]. The anti-apoptotic protein Bcl-2 blocks MOMP, whereas the pro-apoptotic protein Bax activates it [42]. The MOMP causes the release of Cyt c into the cytosol, consequently causing caspase activation and apoptosis [41]. In the present study, MA significantly reduced the expression of Caspase-8, Caspase-9, Bax, and Cyt c, indicating that MA cloud protect hepatocytes from weaning-induced apoptotic death.

When mitochondria are damaged, the leaky electrons from MRC can react directly with oxygen to form endogenous ROS [43]. The MRC II has been reported to play a critical role in mitochondrial ROS generation [44]. The MRC II-mediated ROS production is maximal at low succinate concentration [45], which is in line with our previous study, where we found that W4 has the highest ROS content and the lowest succinate content compared with W0 [18]. In the present study, MA improved the activity of MRC I and MRC IV, indicating that MA decreased endogenous ROS generation possibly by reducing mitochondrial electron leakage from the MRC. Mitochondria are dynamic organelles whose morphology, quality, and abundance are tightly controlled by fusion, fission, biogenesis, and mitophagy [46]. The MFN2 and OPA1 mediate mitochondrial fusion, whereas DRP1 and FIS1 mediate mitochondrial fission [47]. In the present study, the higher expression of DRP1 and lower expression of OPA1 after weaning indicated that mitochondrial fission was promoted and fusion was suppressed at the early stage after weaning. The MA promoted mitochondrial fusion by up-regulating the expression of OPA1 and MFN2.

Mitophagy is one of the main methods used to eliminate damaged mitochondria and the Pink1/Parkin pathway plays a vital role in mitophagy [48]. The Pink1 accumulates on the surface of dysfunctional mitochondria where it recruits and activates Parkin, which triggers various cellular signals and culminates in engulfment of damaged mitochondria within autophagosomes and degradation by lysosomes [49]. The P62 and LC3B are important biomarkers of autophagy. The ratio of LC3B II to LC3B I positively correlates with the number of autophagosomes [50]. The P62 can bind to LC3B and be degraded via autophagy [51]. In piglets, weaning decreases the intestinal expression of Pink1 and Parkin, and increases the ratio of LC3B II to LC3B I [52]. The hepatic ratio of LC3B II to LC3B I and the number of autophagic vacuoles increases 24 h after weaning [53]. The present study showed that weaning increased the expression of Pink1 and ratio of LC3B II to LC3B I, while MA decreased the expression of Pink1, Parkin and P62. These data suggested that MA reduced mitophagy, and it was in line with the results we observed through TEM assay. MicroRNAs (miRNAs) are small regulatory RNAs, which participate in regulation of cell death [54, 55]. Recent studies have implicated miR-421 in various disease [56–59]. The miR-421 can directly regulate the expression of Pink1 [60, 61]. In the present study, we found that miR-421 expression significantly decreased and Pink1 expression significantly increased on W4 compared to those on W0 (Additional file 2: Fig. S1). However, the mechanism by which weaning regulate the expression of miR-421 remains unknown and requires further investigation.

Oxidative stress is a major concern that affects the overall health and productivity of animals in modern production systems, and antioxidants have been widely used to mitigate these detrimental effects [62, 63]. In this study, we observed that MA had great potential to protect piglets from weaning-induced oxidative damage. This suggested that MA could be beneficial not only for weaning piglets, but also for other farm animals. Additionally, increasing evidence indicates that oxidative stress plays a crucial role in the pathogenesis and progression of liver diseases in humans. The application of antioxidants is a good strategy to prevent and treat liver diseases associated with oxidative stress [64, 65]. Our data indicated that MA also offered potential constituents for the research on functional foods and biomedicine. However, a limitation of this study is the lack of knowledge regarding the specific active components and mechanisms of MA. While we analyzed the ingredients of MA [24], further research using network pharmacology, multi-omics, and molecular biology will be conducted to address this gap.

## Conclusions

The present study showed that MA improved growth performance of weaning piglets, and reversed weaning-induced oxidative damage, mitochondrial dysfunction and apoptosis. Our results suggested that MA had a promising prospect for maintaining the liver health in weaning piglets, and provided a reference for the studies of liver diseases in humans.

## Supplementary Information

**Additional file 1: Table S1** Antibodies used in this study.

**Additional file 2: Fig. S1** The relative expression of miR-421. The W0 represented the weaning day and W4 represented 4 d after weaning. Data were presented as mean ± SEM (*n* = 6). *: *P* < 0.05; **: *P* < 0.01. miR-421: microRNA-421.

## Data Availability

Data described in the manuscript will be made available upon request pending.

## Abbreviations

ADFI: Average daily feed intake
ADG: Average daily gain
Bax: Bcl-2 associated X protein
Bcl-2: B-cell lymphoma 2
Caspase-1: Cysteine aspartate-specific protease 1
Caspase-3: Cysteine aspartate-specific protease 3
Caspase-8: Cysteine aspartate-specific protease 8
Caspase-9: Cysteine aspartate-specific protease 9
CAT: Catalase
Cyt c: Cytochrome c
DAPI: 4′,6-Diamidino-2-phenylindole
DRP1: Dynamin-related protein 1
FIS1: Fission protein 1
GPX: Glutathione peroxidase
GSH: Glutathione
H_2_O_2_: Hydrogen peroxide
HSI: Hepatosomatic index
LC3B: Microtubule associated protein 1 light chain 3 beta
MA: Microbe-derived antioxidants
MDA: Malondialdehyde
MFN2: Mitofusin 2
MMP: Mitochondrial membrane potential
MOMP: Mitochondrial outer membrane permeabilization
MRC: Mitochondrial respiratory chain
O_2_^−^: Superoxide anion
OPA1: Optic atrophy protein 1
P62: Sequestosome 1
Parkin: E3 ubiquitin ligase
PC: Protein carbonyl
Pink1: PTEN induced putative kinase 1
RIPA: Radio-immunoprecipitation assay
ROS: Reactive oxygen species
T-AOC: Total antioxidant capacity
T-SOD: Total superoxide dismutase
·OH: Hydroxyl radical
8-OhdG: 8-Hydroxy-2′-deoxyguanosine
