# Correction: Effects of microbe-derived antioxidants on growth performance, hepatic oxidative stress, mitochondrial function and cell apoptosis in weaning piglets

**DOI:** 10.1186/s40104-024-01116-2

**Published:** 2024-11-04

**Authors:** Chengbing Yu, Yuxiao Luo, Cheng Shen, Zhen Luo, Hongcai Zhang, Jing Zhang, Weina Xu, Jianxiong Xu

**Affiliations:** Shanghai Key Laboratory of Veterinary Biotechnology, School of Agriculture and Biology, Shanghai Jiao Tong University, Shanghai, 200240 China

**Correction** **:** **J Animal Sci Biotechnol 15, 128 (2024)**

**https://doi.org/10.1186/s40104-024-01088-3**

Following publication of the original article [1], the authors reported that in the original Fig. 3A, on W14, the pictures stained by DHE and DAPI fluorochrome, and the merged picture were placed out of order.

The original Fig. 3 was:

**Fig. 3:**
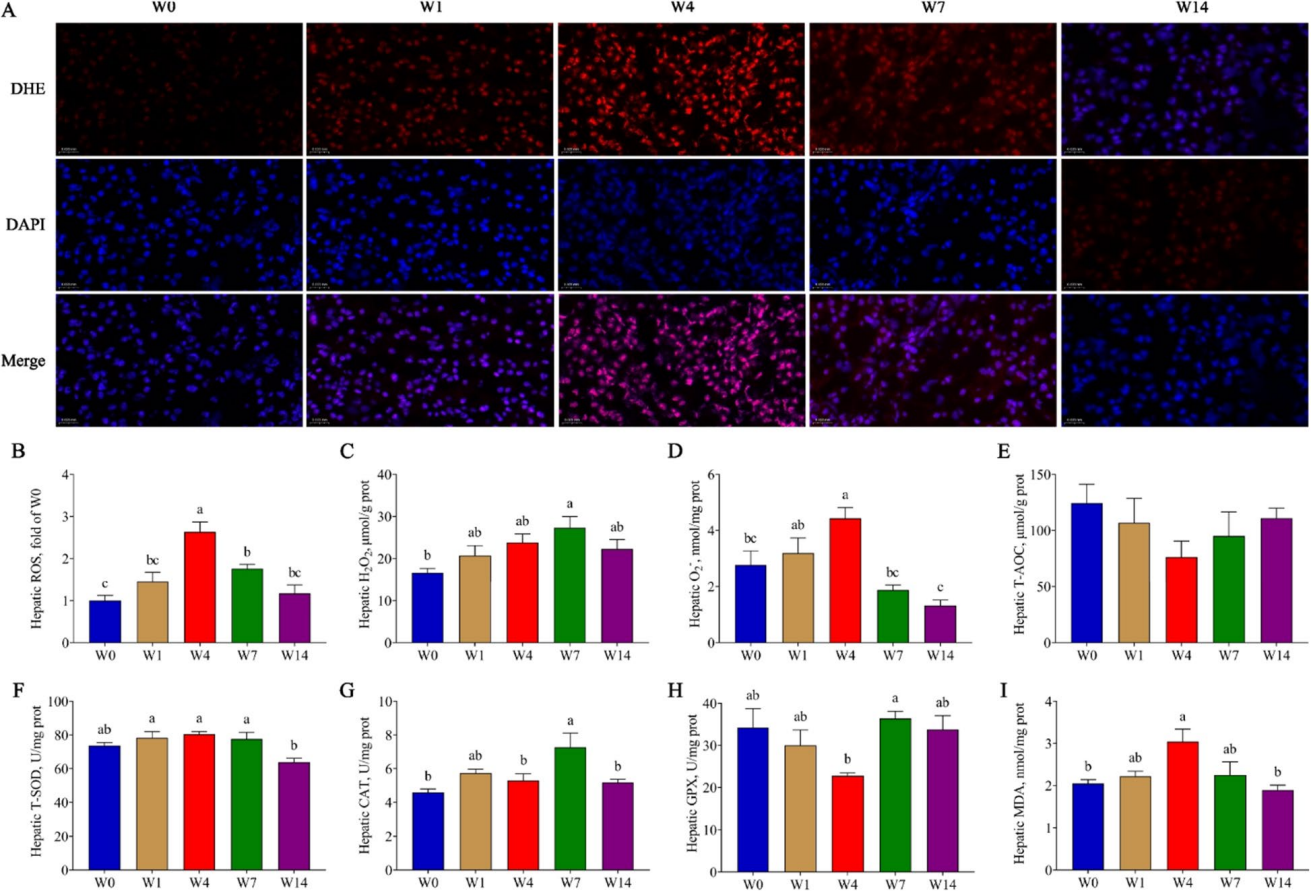
The hepatic redox parameters. **A** and **B** DHE Staining with frozen liver sections and fluorescence intensity of ROS; **C**–**E** Hepatic H_2_O_2_, O_2_^−^ and T-AOC content; **F**–**H** Hepatic T-SOD, CAT and GPX activity; **I** Hepatic MDA content. W0, W1, W4, W7, and W14 respectively represented 21, 22, 25, 28, and 35 days of age. Data were presented as mean ± SEM (ROS, *n* = 3; others, *n* = 6). Values with different letters differ significantly (*P* < 0.05). ROS: Reactive oxygen species; T-AOC: Total antioxidant capacity; T-SOD: Total superoxide dismutase; CAT: Catalase; GPX: Glutathione peroxidase; MDA: Malonaldehyde

The correct Fig. 3 should be:

**Fig. 3:**
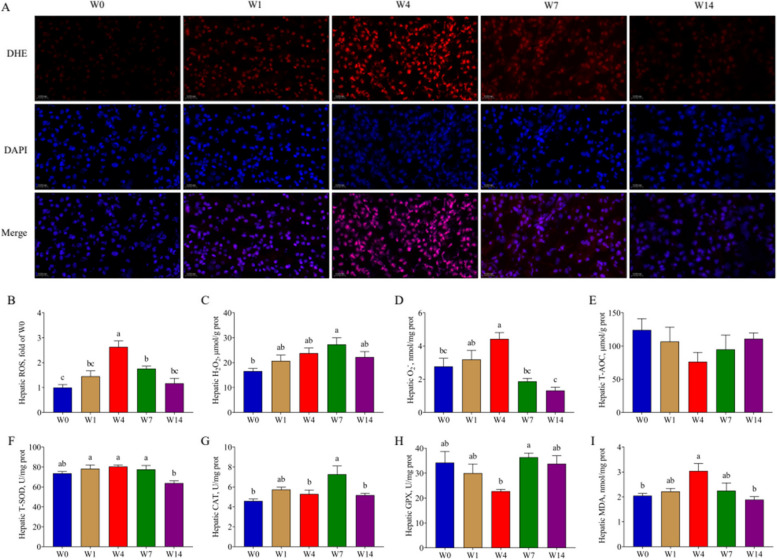
The hepatic redox parameters. **A** and **B** DHE Staining with frozen liver sections and fluorescence intensity of ROS; **C**–**E** Hepatic H_2_O_2_, O_2_^−^ and T-AOC content; **F**–**H** Hepatic T-SOD, CAT and GPX activity; **I** Hepatic MDA content. W0, W1, W4, W7, and W14 respectively represented 21, 22, 25, 28, and 35 days of age. Data were presented as mean ± SEM (ROS, *n* = 3; others, *n* = 6). Values with different letters differ significantly (*P* < 0.05). ROS: Reactive oxygen species; T-AOC: Total antioxidant capacity; T-SOD: Total superoxide dismutase; CAT: Catalase; GPX: Glutathione peroxidase; MDA: Malonaldehyde

The original article [1] has been updated.
