# Correction: Effects of microbe-derived antioxidants on growth performance, hepatic oxidative stress, mitochondrial function and cell apoptosis in weaning piglets

**DOI:** 10.1186/s40104-025-01277-8

**Published:** 2025-09-18

**Authors:** Chengbing Yu, Yuxiao Luo, Cheng Shen, Zhen Luo, Hongcai Zhang, Jing Zhang, Weina Xu, Jianxiong Xu

**Affiliations:** Shanghai Key Laboratory of Veterinary Biotechnology, School of Agriculture and Biology, Shanghai Jiao Tong University, Shanghai, 200240 China

**Correction: J Animal Sci Biotechnol 15, 128 (2024)**

**https://doi.org/10.1186/s40104-024-01088-3**

Following the publication of the original article [1], it is reported that in Fig. 4, the β-actin band in Fig. 4C was mistakenly used as the FIS1 protein band in Fig. 4B. It was merely an error in the use of the band image, and the corresponding statistical values remain correct. Importantly, this error does not affect the results and conclusion of the study.

Incorrect Fig. 4:

**Fig. 4:**
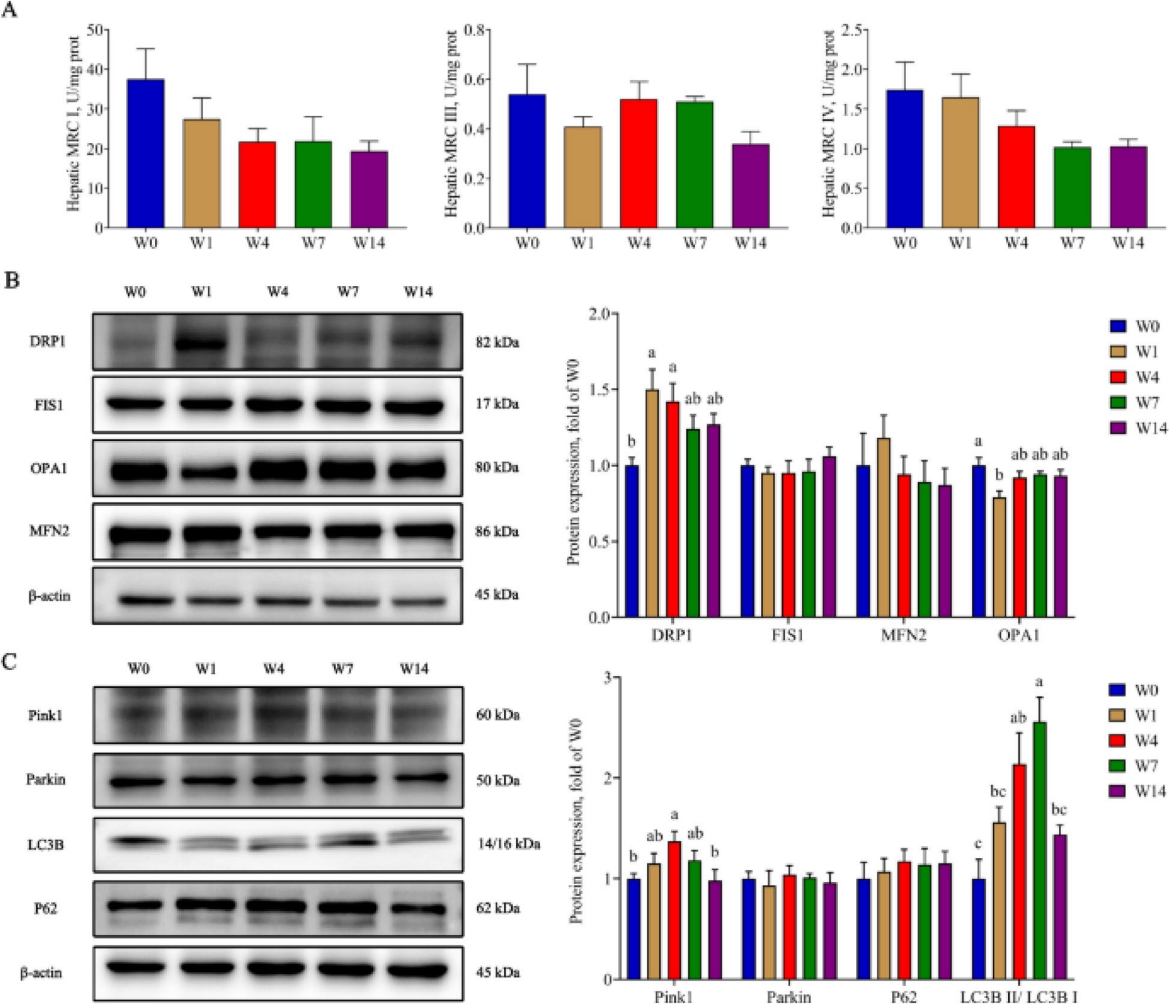
The effects of weaning on mitochondrial function. **A** Activity of mitochondrial respiratory chain complexes; **B** The expression of proteins related to mitochondrial fusion and fission; **C** The expression of proteins related to mitophagy. W0, W1, W4, W7, and W14 respectively represented 21, 22, 25, 28, and 35 days of age. Data were presented as mean ± SEM (*n* = 6). Values with different letters differ significantly (*P* < 0.05). MRC I: Mitochondrial respiratory chain complex I; MRC III: Mitochondrial respiratory chain complex III; MRC IV: Mitochondrial respiratory chain complex IV; DRP1: Dynamin-related protein 1; MFN2: Mitofusin 2; FIS1: Fission protein 1; OPA1: Optic atrophy protein 1; Pink1: PTEN induced putative kinase 1; Parkin: E3 ubiquitin ligase; P62: Sequestosome 1; LC3B: Microtubule associated protein 1 light chain 3 beta

Correct Fig. 4:

**Fig. 4:**
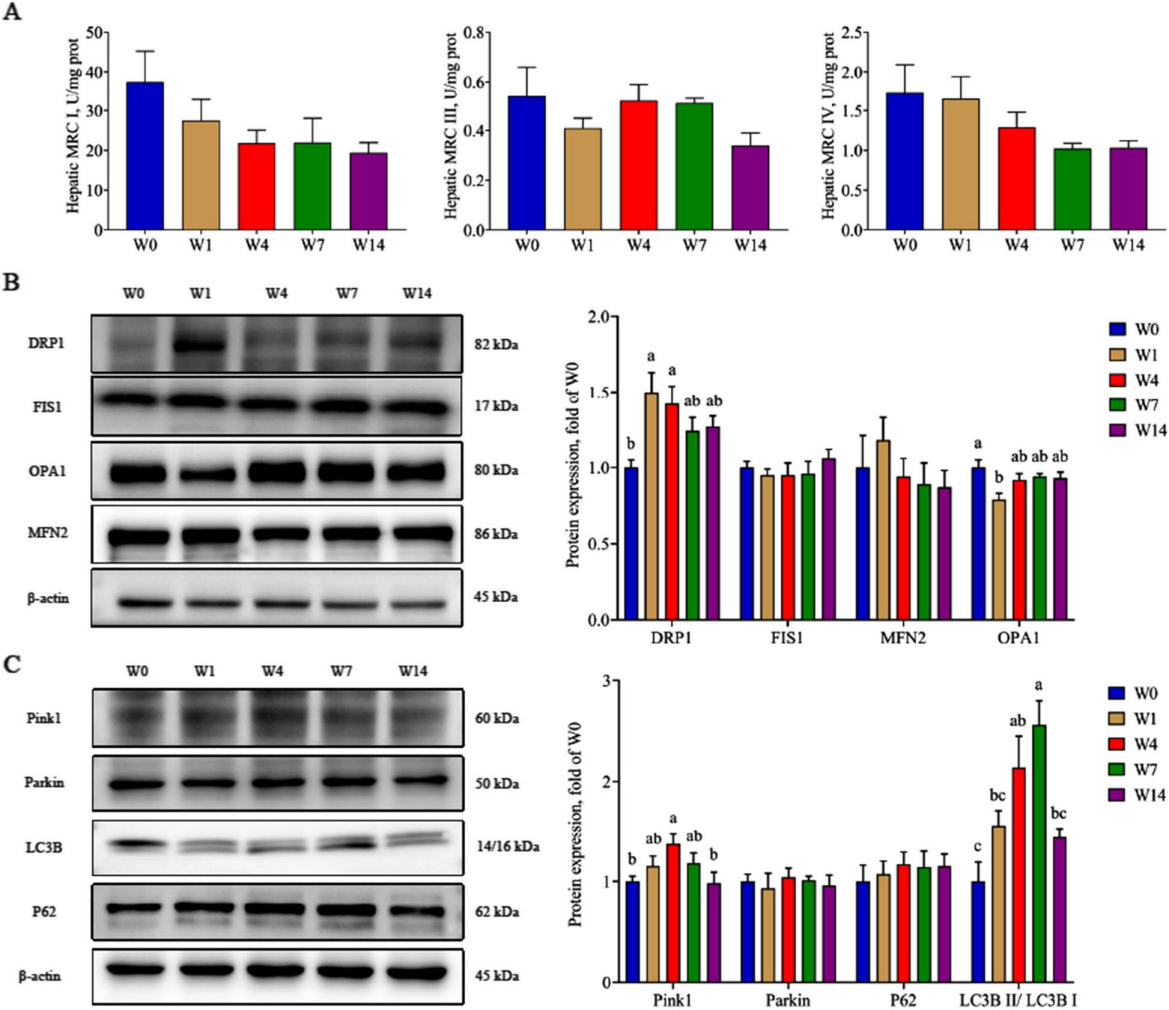
The effects of weaning on mitochondrial function. **A** Activity of mitochondrial respiratory chain complexes; **B** The expression of proteins related to mitochondrial fusion and fission; **C** The expression of proteins related to mitophagy. W0, W1, W4, W7, and W14 respectively represented 21, 22, 25, 28, and 35 days of age. Data were presented as mean ± SEM (*n* = 6). Values with different letters differ significantly (*P* < 0.05). MRC I: Mitochondrial respiratory chain complex I; MRC III: Mitochondrial respiratory chain complex III; MRC IV: Mitochondrial respiratory chain complex IV; DRP1: Dynamin-related protein 1; MFN2: Mitofusin 2; FIS1: Fission protein 1; OPA1: Optic atrophy protein 1; Pink1: PTEN induced putative kinase 1; Parkin: E3 ubiquitin ligase; P62: Sequestosome 1; LC3B: Microtubule associated protein 1 light chain 3 beta

Additionally, in section “MA alleviated the oxidative damage”, “decreased” was mistakenly spelled as “increased” in the sentence: “MDA and 8-OHdG content significantly increased in the MA group compared with the CON group (*P* < 0.05, Fig. 7G and H)”, which should be corrected to: “MDA and 8-OHdG content significantly decreased in the MA group compared with the CON group (*P* < 0.05, Fig. 7G and H)”. The relevant statistical results and descriptions in the discussion section are correct, and this error does not affect the results and conclusion of the study.

The original article [1] has been updated.
