# Supplementary material for: Effects of microbe-derived antioxidants on growth performance, hepatic oxidative stress, mitochondrial function and cell apoptosis in weaning piglets
Source: J Anim Sci Biotechnol. 2024 Oct 2;15:128. doi: 10.1186/s40104-024-01088-3 (PMC11445872; doi:10.1186/s40104-024-01088-3)
Supplement: Supplementary file 1 — Additional file 1: Table S1 Antibodies used in this study. [file 40104_2024_1088_MOESM1_ESM.docx]

Table S1 Antibodies used in this study

| Antibody | Item number | Company |
| --- | --- | --- |
| DRP1 | 26187-1-AP | Proteintech, Wuhan, China |
| FIS1 | 10956-1-AP | Proteintech, Wuhan, China |
| Parkin | 14060-1-AP | Proteintech, Wuhan, China |
| Bax | 50599-2-Ig | Proteintech, Wuhan, China |
| Bcl-2 | 12789-1-AP | Proteintech, Wuhan, China |
| Cyt c | 10993-1-AP | Proteintech, Wuhan, China |
| LC3B | NB600-1384SS | NOVUS, Littleton, CO, USA |
| P62 | NBP1-48320SS | NOVUS, Littleton, CO, USA |
| Caspase-3 | WL04004 | Wanleibio, Shenyang, China |
| Caspase-8 | WL03426 | Wanleibio, Shenyang, China |
| Caspase-9 | WL03421 | Wanleibio, Shenyang, China |
| OPA1 | 80471 | CST, Danvers, MA, USA |
| MFN2 | 9582 | CST, Danvers, MA, USA |
| Pink1 | 6946 | CST, Danvers, MA, USA |
| β-actin | 4970 | CST, Danvers, MA, USA |
| Goat anti-rabbit IgG | 7074 | CST, Danvers, MA, USA |
| Horse anti-mouse IgG | 7076 | CST, Danvers, MA, USA |

DRP1: Dynamin-related protein 1; MFN2: Mitofusin 2; FIS1: Fission protein 1; OPA1: Optic atrophy protein 1; Pink1: PTEN induced putative kinase 1; Parkin: E3 ubiquitin ligase; P62: Sequestosome 1; LC3B: Microtubule associated protein 1 light chain 3 beta.

Caspase-3: Cysteine aspartate-specific protease 3; Caspase-8: Cysteine aspartate-specific protease 8; Caspase-9: Cysteine aspartate-specific protease 9; Bcl-2: B-cell lymphoma 2; Bax: Bcl-2 associated x protein; Cyt c: Cytochrome c.
