# Supplementary material for: Effects of microbe-derived antioxidants on growth performance, hepatic oxidative stress, mitochondrial function and cell apoptosis in weaning piglets
Source: J Anim Sci Biotechnol. 2024 Oct 2;15:128. doi: 10.1186/s40104-024-01088-3 (PMC11445872; doi:10.1186/s40104-024-01088-3)
Supplement: Supplementary file 2 — Additional file 2: Fig. S1 The relative expression of miR-421. The W0 represented the weaning day and W4 represented 4 d after weaning. Data were presented as mean ± SEM (n = 6). *: P < 0.05; **: P < 0.01. miR-421: microRNA-421. [file 40104_2024_1088_MOESM2_ESM.docx]

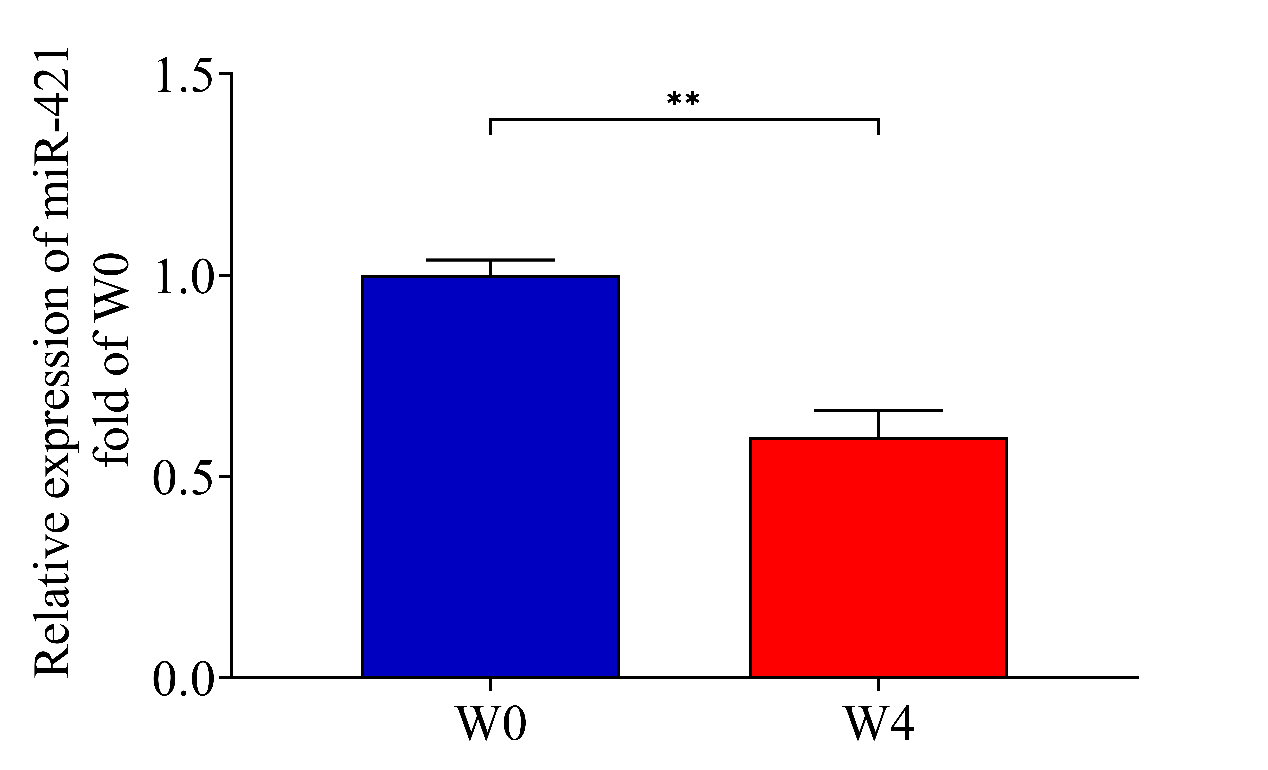


Fig. S1 The relative expression of miR-421. The W0 represented the weaning day and W4 represented 4 days after weaning. Data were presented as mean ± SEM (n = 6). *: *P* < 0.05; **: *P* < 0.01. miR-421: microRNA-421.
